# Morphometrics and behavior of a wild Asian elephant exhibiting disproportionate dwarfism

**DOI:** 10.1186/1756-0500-7-933

**Published:** 2014-12-19

**Authors:** Shermin de Silva, U Sameera Weerathunga, Tennekoon V Pushpakumara

**Affiliations:** Uda Walawe Elephant Research Project, EFECT, Colombo, Sri Lanka; Department of Fish, Wildlife & Conservation Biology, Colorado State University, Fort Collins, CO 80526 USA

**Keywords:** Achondroplasia, Chondrodysplasia, *Elephas maximus*, Dwarf

## Abstract

**Background:**

Dwarfism is a condition characterized by shorter stature, at times accompanied by differential skeletal growth proportions relative to the species-typical physical conformation. Causes vary and are well-documented in humans as well as certain mammalian species in captive or laboratory conditions, but rarely observed in the wild.

**Case presentation:**

We report on a single case of apparent dwarfism in a free-ranging adult male Asian elephant in Sri Lanka, comparing physical dimensions to those of other males in the population as well as in previous literature. The subject M459 was found to have a shoulder height of approximately 195 cm, is shorter than the average height of typical mature males, with a body length of 218 cm. This ratio of body length to height deviates from what is typically observed, which is approximately 1:1, but was similar to the attributes of a dwarf elephant in captivity documented in 1955. We report on behavior including the surprising observation that M459 appears to have a competitive advantage in intrasexual contests. We discuss how this phenotype compares to cases of dwarfism in other non-human animals.

**Conclusion:**

M459 exemplifies a rare occurrence of disproportionate dwarfism in a free-ranging wild mammal that has survived to reproductive maturity and appears otherwise healthy.

## Background

Reduced stature is a shared attribute among individuals exhibiting various forms of dwarfism, though this alone is not diagnostic of dwarfism. Dwarfism may be divided into two types: proportionate, whereby individuals exhibit growth reduction while maintaining isometric proportions relative to the typical, and disproportionate, whereby growth disturbance results in physical proportions that are scaled allometrically. There seems to be little agreement on the scientific criteria, phenotypic attributes, or physiological mechanisms which define proportionate dwarfism in humans, though these terms are applied to individuals from domesticated species such as cattle [1, 2]. Disproportionate dwarfism (variously classified as achondroplasia, chondrodysplasia or diastrophic dysplasia) on the other hand, involves both shorter stature as well as changes to the proportions of the limbs, head and torso [3].

**Figure 1:**
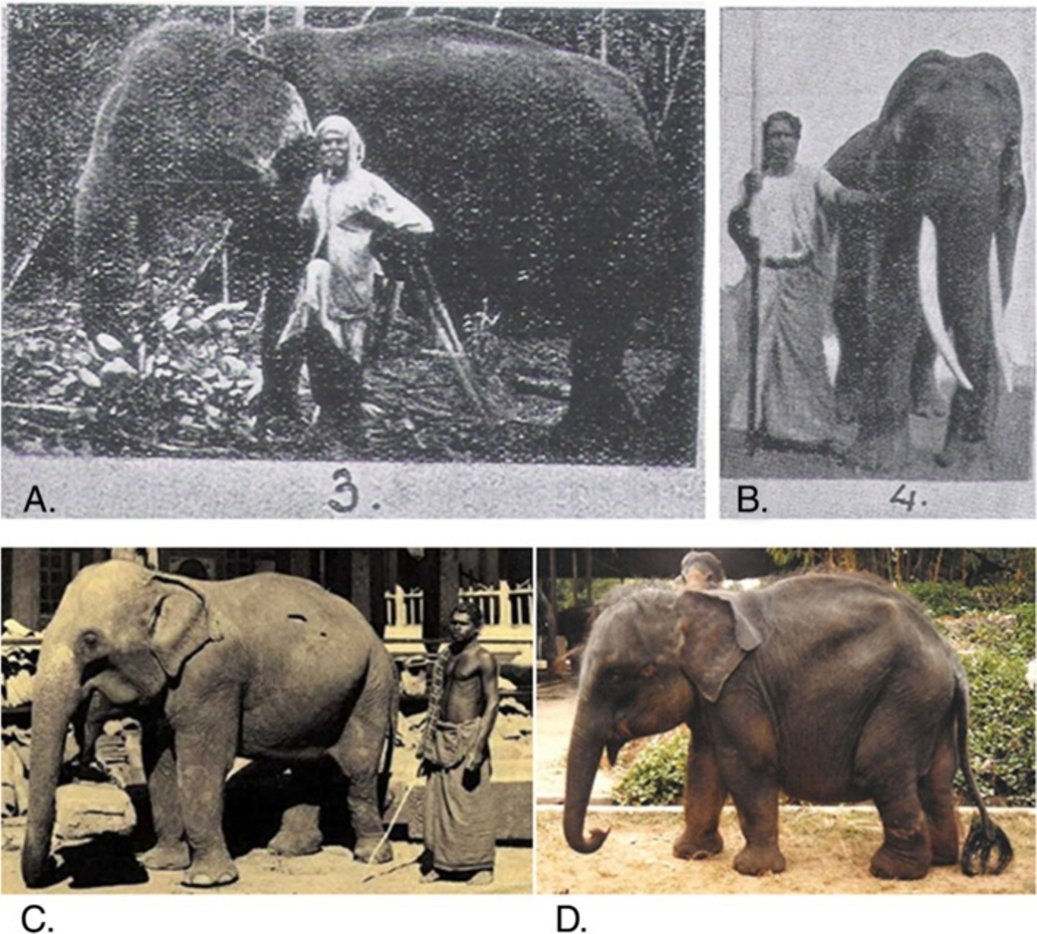
**Dwarf elephants documented in captivity.** Panels **(A)** and **(B)** are reproduced from Deraniyagala 1955, plate 18; **A)** A 35-year-old female described as a dwarf, owned by a person in Sri Lanka; **B)** A 21-year-old male tusker captured in Mannar (north-west Sri Lanka), also described as a dwarf, with some physical measurements provided (see Table 1 and text); **C)** a photograph from Old Ceylon Page of a male elephant kept by the Temple of the Tooth in Kandy; **D)** “Mali” a wild-born female brought to the Dehiwala Zoo, reportedly found in the Anuradhapura area, with an estimated birth date of 1980. Photographed by W. Jackson (Elephant Encyclopedia and Database). It is not clearly established whether any of these cases constitute hereditary dwarfism, as opposed to other mechanisms such as stunted growth through nutrient deficiency or captive confinement.

Dwarfism in non-human animals in the wild has rarely been documented. Among elephants kept in captivity, dwarfism has been suggested in at least four cases, two males and two females (Figure 1). But because measurements and further details are unavailable, it cannot be established whether they are actually dwarfs, or what sort of dwarfism they might represent (see Results). Anecdotal historic accounts exist of apparent dwarfism occurring in the wild in various parts of this species’ range [4], however these are not substantiated. Here we report on the case of dwarfism exhibited by a fully-grown male Asian elephant (*E. maximus maximus*) in Uda Walwe National Park, Sri Lanka [5]. Clinical examination and invasive procedures are precluded as the subject is a free-living animal that is a member of an endangered species. We provide a description which may nevertheless be a useful record of this rare phenomenon, and a comparison to the species-typical phenotype as well as the cases from the prior historical accounts.

## Case presentation

Uda Walawe National Park (UWNP) is located in south-central Sri Lanka (latitudes 6°25’–6°34’N/80°46’–81°00’E). Vehicle-based observations were made during ongoing studies of this elephant population conducted since 2006. All individuals are photographed upon encounter, and individual-identification files based on natural features are maintained. The elephant population occupying UWNP numbers between 800–1200 [6], with a high degree of seasonality in use. In particular, males are known to range beyond this protected area, which is partially encircled by electric fencing.

**Figure 2:**
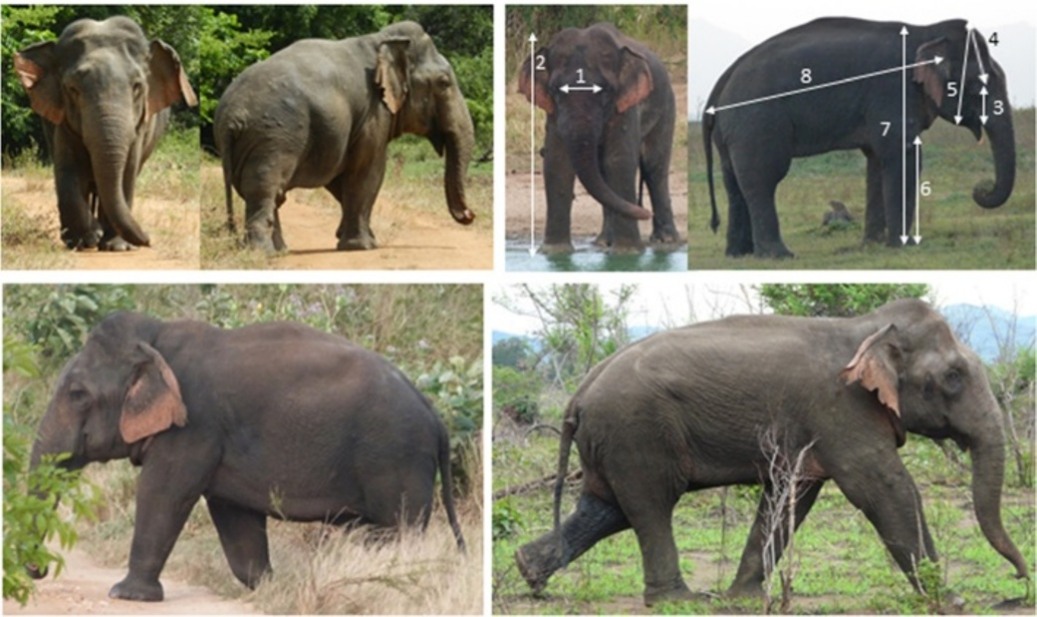
**Dwarf M459 and wild type adult male M054.** M459 (left) proposed dwarf, compared to M054 (right) a typical mature adult male. Numbers indicate ratios given in Table 1 as follows: 1) Width between the eyes; 2) Head to foot; 3) Eye to tusk sheath; 4) Top of skull to eye; 5) Top of skull to chin; 6) Foreleg length; 7) Shoulder height; 8) Body length from base of tail to ear canal. Upper left photographs of M459 courtesy of Ms. C.E. Webber.

**Figure 3:**
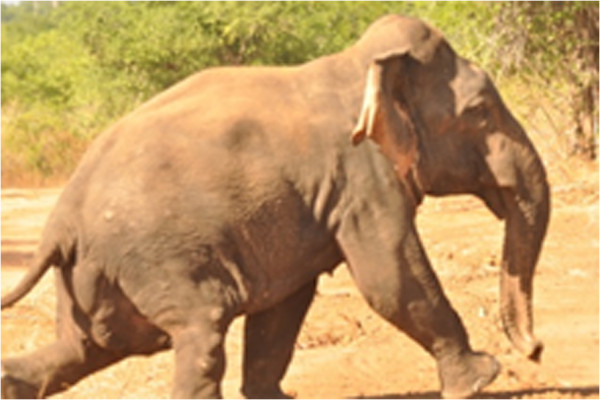
**Dwarf M459, July 12th 2012.** Subject shows no visible signs of musth, but swellings on the rear limbs similar to those observed in 2013 (Figure 2) are evident, indicating that these are old. Photographs courtesy of Dr. N. Jayasena.

The subject was observed by Ms. C.E. Webber, USW and TVP on July 11th 2013, video recorded, and assigned the ID M459 (Figure 2). A video clip of M459 during this encounter may be viewed [7]. It was later found that M459 had also been sighted inside Uda Walawe National Park exactly one year prior, on 12th July 2012 by Dr. N. Jayasena and Ms. F. Hua (Figure 3). He had not been identified in preceding years of the research project and therefore appeared to be a relatively new arrival to UWNP. In 2013 visible temporal secretions indicated he was in the physiological state known as ‘musth’, [5] which is a period of heightened sexual activity and aggression [8], which establishes that M459 is a sexually mature individual that is likely to be over 20 years old. No such sign was visible the previous year. Urine dribbling, which can also be an accompanying signal, was not observed in M459. Swellings on the hind limbs of the subject resemble gunshot wounds (Figures 2 and 3), similar to those seen on the forelimbs of M054 (wild type specimen) on some occasions (Figure 2, upper panels). M459 exhibited no unusual behavior but appeared unhabituated and fearful of observers. Because M459 was not observed in prior years, it is likely that he was a relatively recent occupant of the area.

Shoulder height measurements were made opportunistically using one of two methods. The footprint circumference for a young male exemplifying average stature in this population was measured and the height was calculated as twice this circumference. This ratio appears to remain constant throughout an individual’s life [9]. The height of the subject was measured by proxy using a tree against which he had been standing.

In order to compare the subject’s physical stature to those of typical bulls, we assessed these proportions using photographs of known individuals (Figure 2). We used two views, anterior and lateral. The anterior view was used to determine a single ratio: height from top of head to toes vs. width of the distance between the eyes. The lateral view was used to calculate multiple ratios from the same photograph: the distance from the top of the skull to the eye, the eye to the base of the upper lip (the point where tusks or tushes would protrude), the length of the head, the length of the foreleg, shoulder height and back length measured from ear canal to the base of the tail. Only photographs in which subjects were directly facing or perpendicular to the angle of view of observers were used to limit angular distortions in measurements. Distortions from changes in elevation were minimized by always taking photographs from the same vehicle-based vantage point. Note that we focus on relative proportions rather than absolute measures for comparative purposes.

Morphometric details for the subject and comparative data from other individuals are provided in Table 1. The head is large relative to the length of the foreleg (1:0.91 in m459 whereas wild type ranges from 1:1.09-1:1.40) but the skull itself is proportioned normally compared to other individuals. M459’s estimated shoulder height was approximately 195 cm with a body length of 218 cm. By comparison M343, a young adult male in this population, had already attained a shoulder height of 224 cm (fore and hind foot circumference both equal to 112 cm) and older, larger males are observed, such as M054. The subject is also well below the asymptotic height (~250 cm, range: 220-270 cm) of Asian elephants measured elsewhere in the wild and captivity [9]. M459 exhibits shorter limbs, and slightly shorter height relative to body length to other mature males in this population (Table 1, ratios in column 7 vs. 8). The difference is small (subject shows 1:1.09 whereas most common is 1:1 with a minimum of 1:0.92 and a maximum of 1:1.04 for N = 20 individuals), perhaps because the body itself seems slightly compressed, but we cannot demonstrate this.

**Table 1:** **Relative proportions of dwarf and wild type adult male Asian elephants in Sri Lanka**

	Anterior view		Lateral view					
ID	1. Width between eyes	2. Head to foot	3. Eye to lip	4. Eye to top of skull	5. Head	6. Foreleg	7. Shoulder height	8. Body length
M459	1	3.33	2	3	4.7	4.3	9	9.8
21-yr tusker	1	4.23	-	-	-	-	12	13
M072	1	4.33	2	2.5	4.3	6	11	11
M025	1	4.5	2	3	5	5.5	11	11
M180	1	4.5	2	3	5	6	11	11
M074	1	4.16	2	3	4.7	5.5	11.5	12
M098	1	4.33	2	3	5	6	11.5	11.5
M002	1	4.4	2	3	5	6.2	11.5	11.5
M029	1	4.5	2	3	4.6	5.5	11.5	11.5
M175	-	-	2	2.7	5	6.5	11.5	11.5
M054	1	3.93	2	3.3	5.2	6.2	12	12
M034	1	4.27	2	3	5	6.5	12	-
M023	1	4.57	2	3	5	5.5	12	12
M077	1	5	2	3	5	5.5	12	12
M343	-	-	2	3	5	6.5	12	12
M038	1	4.47	2	3.3	5.5	6	12.5	12.5
M012	1	4.67	2	3.3	5.5	6.5	12.5	12.5
M036	1	4.93	2	3.3	5.2	6.5	12.5	11.5
M030	1	5.17	2	2.8	4.8	6	12.5	11.5
M085	1	4	2	3.5	5.4	6	12.8	13
M260	1	3.67	2	3	4.5	6	13	13
M109	1	4	2	3.5	5.5	6	13	13.5

M343, M054, M002 and M034 are typical males; M459 is the subject exhibiting dwarfism; the 21-yr tusker refers to the individual shown in Figure 1b, putatively also a dwarf (see text). See Figure 2 for corresponding photographs. Relative proportions are standardized across individuals with respect to the smallest measurements in each view: the width between the eyes (anterior) and the distance from the eye to the upper lip (lateral).

We now compare M459 to the prior historical anecdotes of dwarfism (Figure 1). The individuals in Figure 1a and c had no accompanying records of height or body proportions. Visual inspection of Figure 1a suggests that the female in question is likely to be taller than M459 as she is appreciably taller than her handler, but since the height of the latter is unknown this cannot be established. Mali (Figure 1d), a young female rescued from the Anuradhapura area and brought to the Dehiwala Zoological Gardens, appears to exhibit some growth limitation. In all of these cases, it is not clear whether this is a hereditary condition or a result of nutritional deficiency during some critical period. The latter could result in stunted growth, but not qualify as dwarfism. The young tusker captured from the wild in Sri Lanka in 1933 (Figure 1b, Table 1) is a candidate for the first known case of dwarfism and is the only instance for which measurements are available. This individual was stated to have a shoulder height of 183 cm and a back length of 198 cm (measured from behind the ear rather than from the ear canal, to the base of the tail). This height is shorter than the expected height at this age based on the documented literature [9], which should be ~245 cm. However, from the anterior view proportions of the young tusker are more similar to wild type individuals than to M459 (Table 1), while the height is again slightly shorter than back length. Therefore the former may represent a case of proportionate dwarfism. Additionally, while the absolute height of this tusker was shorter relative to the asymptotic height, it was still substantially taller than M459. M459 therefore remains a unique case of disproportionate dwarfism distinct from any others previously documented.

### Behavior

**Figure 4:**
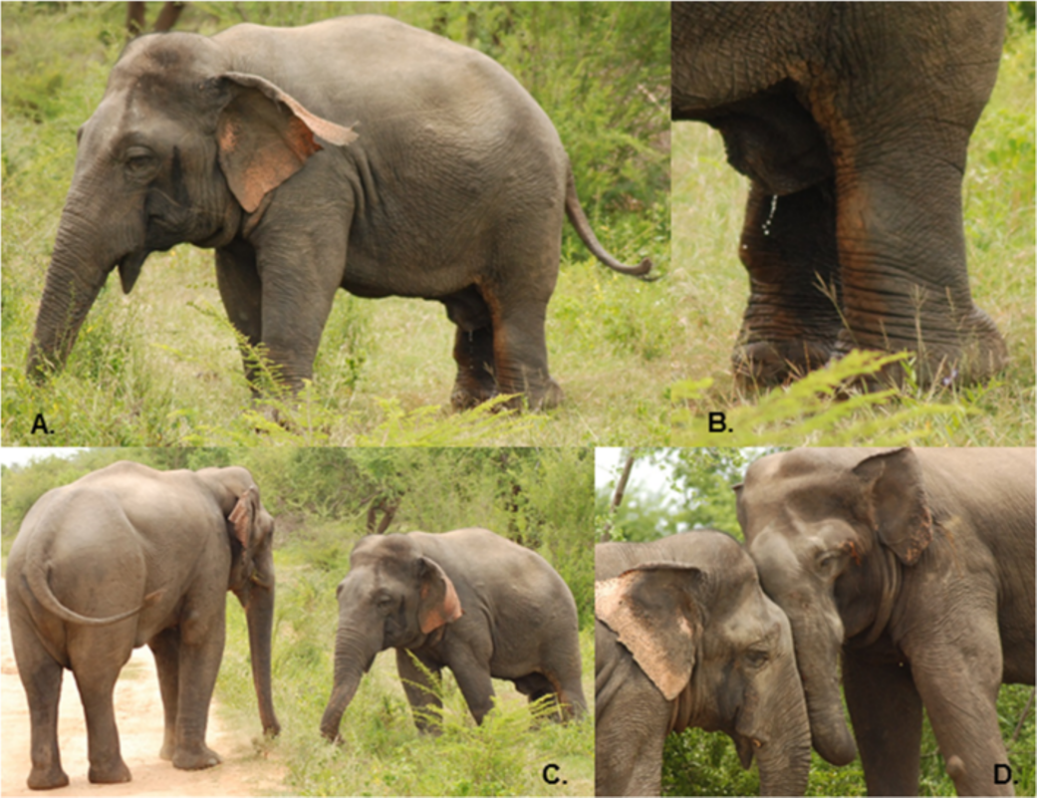
**M459 with M175, 16 June 2014. A**. M459 showing both temporal secretion and urine dribbling. **B**. Urine dribbling. **C**. M459 standing near M175. **D**. Head-to-head contest showing similarity of head proportions and difference in height.

**Figure 5:**
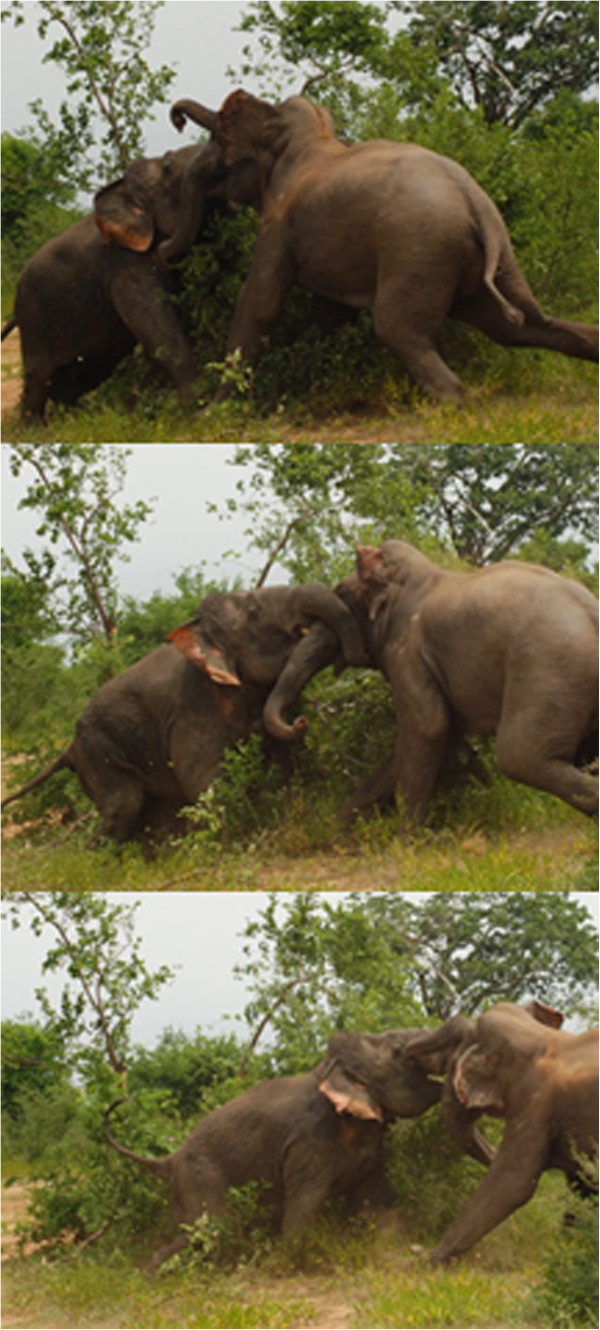
**M459 fighting M175.** This sequence shows M459 forcing M175 backward. Video can also be viewed [10].

Aggression by M459 toward other males was observed in 2013 [5], evidencing apparently normal sexual activity. On June 16 2014, the third year M459 has been seen in Udawalawe, we witnessed a full-scale contest between M459 and M175, a full-sized reproductively mature bull who was also showing signs of being in early musth (Figures 4 and 5). Contests between bulls can last over multiple days, with intermittent rests and changes of pace. We therefore did not observe the entirety of the episode, however we were able to witness interactions over several hours. We noted with surprise that M459 almost always initiated confrontation by moving toward, lunging or chasing M175. M175 initially faced M459 and responded in kind but after several hours exhibited clear signs of losing, moving away and occasionally running to avoid M459 (see video [10]). The pace of interactions was therefore set by M459, whose shorter stature actually appeared to be advantageous in the head-to-head combat style of bull elephants (Figure 4) as it facilitated throwing his bulk directly at an opponent, whereas a taller individual had to stoop down awkwardly and risk falling (Figures 4 and 5). We did not observe the outcome of this interaction. Two days later on June 18 we observed M459 at the same location, with a group of females and calves. He rested beneath the same tree as the group and moved with it, behaviors that can accompany mate guarding. Mating is generally preceded by intense competition, in which females actively run away from potential suitors, sometimes over multiple days. It is not known whether M459 could chase and physically mount a female in oestrus as this has not yet been observed.

### Disproportionate dwarfism in other species

In humans disproportionate dwarfism arises most commonly due to a single amino acid mutation in the protein known as fibroblast growth factor receptor 3 (FGFR-3), which can limit bone growth [3, 11, 12]. However, dwarfism can manifest from several distinct physiological mechanisms, only some of which have identified and heritable genetic components. For instance, mutations in natriuretic peptide receptor 2 (Npr2) [13, 14], or COL11A2 and ACAN, both of which are involved in the development of cartilage [15, 16].

The mechanisms of some cases remain as yet unknown. Friesian horses, for instance, exhibit a form of dwarfism in which both the fore and hind limbs are approximately 25% shorter relative to the breed’s typical conformation, with relatively larger heads, longer hooves and hyperextension of the fetlocks [17]. The head proportions themselves appear relatively normal, similar to what is observed in M459, unlike the effects observed with disruptions to FGFR1 or FGFR2, two candidate genes found within a region of the genome that appears to be highly associated with the dwarf phenotype in this breed [18]. Nor do the documented cases resemble phenotypes resulting from disturbance to PROP1, another candidate gene within the same region, that regulates growth hormone production. Thus the causal mechanism remains an open question even in these clinically-documented studies. Since growth disturbances can clearly arise from a wide array of mechanisms, as close clinical study of this free-ranging individual are not possible, the specific cause of the dwarfism exhibited by M459 cannot be conclusively established.

Among non-domesticated species housed in captivity, congenital anomalies have been observed in individuals from two species of tamarins (*Sanguinus oedipus* and *Sanguinus fuscicollis*) resulting from matings between siblings. Both were either stillborn or died as neonates [19]. These cases may have resulted from the unnatural housing conditions which restricted movement therefore it is unknown whether such cases would ever occur in the wild. At this time, we are aware of only one other case report documenting naturally-occurring disproportionate dwarfism in the wild, which was of a single female dwarf red deer (*Cervus elephus*), culled from Argyll, Scotland [20]. The paucity of documented cases of disproportionate dwarfism in the wild may be due to reduced fitness and survival as a consequence of one or more genetic abnormalities. Individuals exposed to natural predators would likely not survive long enough to be observed. Because Asian elephants typically have few predators other than humans, as well as being associated with vigilant social groups during early development, the survival probability of individuals such as M459 may be enhanced. Such cases may also be rarely documented due to publication biases resulting from the lack of venues for reporting them.

## Conclusions

The subject M459 exhibits allometric differences in physical dimensions relative to other adult males, primarily shorter height relative to head size and body length. He moreover shows obvious signs of sexual maturity, most notably temporal secretions indicative of musth. He appears otherwise healthy, in good body condition and behaviorally normal. M459 therefore represents a rare instance of naturally-occurring disproportionate dwarfism in a wild free-ranging individual.

## Consent

This work was conducted the approval of the Institutional Animal Care and Use Committee of Colorado State University, protocol no. 11-2816A and with approval of the Department of Wildlife Conservation (DWC), Sri Lanka.
